# Epidemiological and Molecular Characteristics of the PB1-F2 Proteins in H7N9 Influenza Viruses, Jiangsu

**DOI:** 10.1155/2015/804731

**Published:** 2015-01-20

**Authors:** Pingmin Wei, Wei Li, Hairong Zi, Michael Cunningham, Yan Guo, Yang Xuan, Taha Hussein Musa, Pengfei Luo

**Affiliations:** ^1^Key Laboratory of Environmental Medicine Engineering, Ministry of Education, School of Public Health, Southeast University, Nanjing, Jiangsu 210009, China; ^2^Department of Infectious Disease Prevention and School Health, Nanjing Municipal Center for Disease Control and Prevention, 2 Zizhulin, Nanjing, Jiangsu 210009, China; ^3^Department of Chronic Non-Communicable Disease Control and Prevention, Jiangsu Provincial Center for Disease Control and Prevention, Nanjing, Jiangsu 210009, China

## Abstract

The recent sporadic infections of humans in China with previously unrecognized avian influenza A virus of the H7N9 subtype (A(H7N9)) have caused concern. The aim is to find out the epidemiological and molecular analysis of the PB1-F2 proteins in H7N9 influenza viruses, in Jiangsu province. Sequences were obtained from GISAID database. Data were analyzed by using Molecular Evolutionary Genetics Analysis software and Bayesian Markov chain Monte Carlo method. From March 1, 2013, to May 31, 2014, 53 patients were confirmed to be infected with the H7N9 virus; one was a retrospective case in Jiangsu province. 38 sequences of PB1 in H7N9 of Jiangsu were obtained from the GISAID online and were then divided into three lineages. Of these sequences, 4 sequences and 3 sequences encode an N-terminally truncated PB1-F2 (52aa)polypeptide and C-terminally truncated PB1-F2 (76aa) polypeptide, respectively. The remaining sequences encode a full-length PB1-F2 (90aa). We estimated a mean evolutionary rate of 3.053 × 10^−3^ subs/site/year (95% HPD: 2.021 × 10^−3^–4.051 × 10^−3^). The site-by-site analysis of selection pressure analysis revealed positively and negatively (12, 3), respectively, selected sites. Influenza A (H7N9) virus adapting into new host, PB1-F2 of H7N9, might be faced with higher selection pressures.

## 1. Introduction

During February and March 2013, a novel influenza A (H7N9) virus occurred in Shanghai, China. Then in Anhui, Jiangsu, Zhejiang, and other provinces more and more cases were detected and most patients became severely ill with deadly results [1]. As of May 31, 2014, a total of 332 patients were reported to have suspected H7N9 virus and 130 of them died. Influenza H7N9 is one of a subgroup of influenza A viruses that has not been detected in humans. Virologists, biologists, and epidemiologists immediately carried out new research, quickly identifying the new virus. Sequencing analyses revealed that the 8 genes from the virus were of avian origin, with the 6 internal genes coming from the avian influenza A (H9N2) virus; the PB1, NP, M, and NS of the new virus are from chicken-borne viruses, which have recently been circulating in poultry in East China [2]. Because the new virus (H7N9) was never recognized in humans before, there are many urgent questions and global public health concerns [3].

Chen and colleagues reported that they discovered a novel protein (PB1-F2) encoded by an open reading frame (ORF) lurking in an alternative reading frame of segment 2 that is not essential for viral replication* in vitro*. They demonstrated that the protein is mainly located in cell mitochondria, can be recognized by the host's immune cells (CD8 + T cells), and has a role in modulating the host response by hastening the death of immune cells [4]. And recent research shows that its contribution to the pathogenicity and the genetic evolutionary characteristics of influenza A virus (IAV) has become one of the hottest topics in the study of genetic evolution of IAV. PB1-F2 has been implicated in the regulation of polymerase activity, immunopathology, susceptibility to secondary bacterial infection, and the induction of apoptosis.

When PB1-F2 undergoes oligomerization it is mediated by two distinct domains located in the N- and C-terminal, respectively [5]. A region near the C-terminal of PB1-F2 is necessary and sufficient for achieving inner mitochondrial membrane localization, PB1-F2 interacts with the adenine nucleotide translocator 3 (ANT3) and the voltage-dependent anion channel 1 (VDAC1) proteins of the permeability transition pore complex (PTPC) at the inner and outer mitochondrial membranes [6], and ANT3 and VDAC1 are believed to interact, forming the PTPC, which leads to dissipation of the inner mitochondrial membrane potential and the release of apoptotic mediators from the mitochondrial intermembrane space [7–9]. In this way, apoptosis is induced by mitochondrial pathway. Moreover, PB1-F2-knockout influenza virus induced less cell death than the wild-type virus in the mouse model, illustrating the role that PB1-F2 may play in promoting the apoptosis.

PB1-F2 does not exclusively locate in the mitochondria but is also found in the nucleus; Mazur and colleagues demonstrated that PB1-F2 indirectly regulates polymerase activity through its interaction with PB1 variants of A/Puerto Rico/8/34 (H1N1), resulting in enhanced polymerase activity [10]. Moreover, a lack of PB1-F2 during infection resulted in a decreased viral polymerase activity, which produced a smaller plaque phenotype in MDCK cells compared to the wild type [10]. Košík and colleagues also found that the N-terminal region of the PB1-F2 protein was responsible for the increase in PB1 protein expression [11]. Mcauley et al. carried out studies on other influenza A viruses, including H1N1 (1918), H1N1 (1956), H3N2 (1995), and H5N1 (2004), and they also demonstrated that PB1-F2 colocalizes with PB1 and enhances polymerase activity [12]. The authors suggested that the colocalization of PB1-F2 with PB1 most likely leads to retention of PB1 in the nucleus in the late phase of replication and that its function is regulated by PKC-mediated phosphorylation [13].

Influenza mortality includes deaths from the primary viral infection as well as from secondary bacterial pneumonia. And the major cause of death is secondary bacterial pneumonia. During influenza virus infection, loss of ciliated and mucus-producing epithelial cells occurred [14] and, due to this, the function of clearing microorganisms and particulate matter is disrupted, so conditioned pathogens can stay in the lung longer and in turn cause a secondary infection [15]. In addition, another possible reason for secondary bacterial infection is the removal of alveolar macrophages by PB1-F2 protein. Alveolar macrophages interact with inhaled microorganisms and particulate matter, playing an important role in immunity and lung defense [16]. During an IAV infection, any damage to alveolar macrophages by PB1-F2 could lead to a bacterial infection in the lungs. So with the removal of alveolar macrophages by the PB1-F2 protein, the induction of the acquired immune response is delayed and impaired. This allows for a reduced clearance function and promotes secondary bacterial pneumonia [15].

## 2. Methods

In our study, we analyzed the epidemiological and molecular characteristics of the PB1-F2 proteins of novel influenza A (H7N9) virus in Jiangsu province. All sequences were obtained from the Global Initiative on Sharing Avian Influenza Data (GISAID) database, from January 1, 2013, and accessed on May 31, 2014. The IRD was queried to return all available sequences for the PB1-F2 protein of H7N9 strains. Sequences were aligned using the multiple sequence alignment (MSA) tool MUSCLE. Nucleotide distributions at amino acid sites 62, 66, 75, 79, and 82 of the PB1-F2 and phylogenetic tree were analyzed using Molecular Evolutionary Genetics Analysis software (MEGA 5.2).

We used the Bayesian Markov chain Monte Carlo method, implemented in BEAST package (version 1.8.0), to jointly estimate phylogenies, divergence times, and other evolutionary parameters for PB1-F2. The General Time-Reversible (GTR) model was used with a gamma parameter of 4 and invariant sites.

To identify the existence of positive selection pressure at the whole-gene level as well as the individual codon sites, we estimated the mean numbers of nonsynonymous substitutions (dN) and synonymous substitutions (dS) per site (ratio dN/dS) and three likelihood methods were used (SLAC, FEL, and REL) [17, 18]. The dN/dS estimates were based on neighbor-joining (*NJ*) trees under the HKY85 substitution model, through the Datamonkey web-based interface (http://www.datamonkey.org).

## 3. Results

### 3.1. Epidemiology of H7N9 in Jiangsu Province

**Figure 1:**
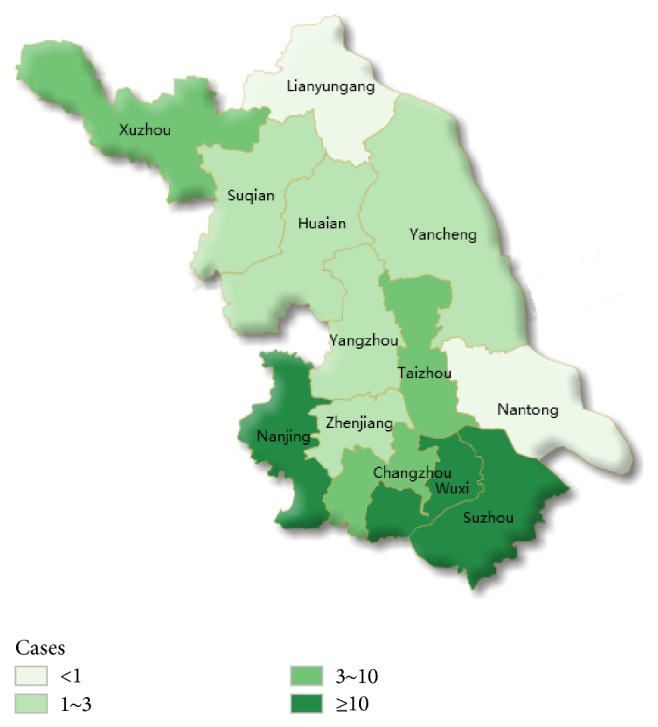
Geographic distribution of H7N9 cases in Jiangsu province.

From March 1, 2013, to May 31, 2014, 53 patients were confirmed to be infected with the H7N9 virus and one of them was retrospective case in Jiangsu province. Cases were identified in the following municipalities: Nanjing (16 confirmed cases), Suzhou (10 cases), Wuxi (10 cases), Changzhou (4 cases), Xuzhou (3 cases), Taizhou (3 cases), Huaian (2 cases), Yancheng (2 cases), Zhenjiang (1 case), Yangzhou (1 case), and Suqian (1 case) and most of the cases (40/53) were located in southern Jiangsu (Figure 1) and were aggregated in the period from late March to early April 2013. Cases were predominantly male and older, aged from 15 to 85 years; the median age of patients with H7N9 virus infection was 54.0 years. Cough and fever were the most common clinical symptoms. Except for one family cluster that has been identified almost all cases have been sporadic; evidence does not support sustained human-to-human transmission of influenza H7N9 virus [19]. Of 527 close contacts, all of these symptomatic patients tested negative for H7N9. After closure of live poultry markets in April, cases greatly decreased or disappeared [20].

### 3.2. Bayesian Markov Chain Monte Carlo Evolutionary Analyses

To elucidate the potential adaptive mutation of the PB1-F2 protein of H7N9 virus, we compared the H7N9 PB1-F2 with H9N2 PB1-F2 at the amino acid level. A strict molecular clock with an exponential growth model was used. For each analysis, a chain length of 10,000,000 was used and sampled every 10,000 states. Convergence was confirmed with Tracer v1.6. Under this condition, we estimated a mean evolutionary rate of 3.053 × 10^−3^ subs/site/year (95% HPD: 2.021 × 10^−3^ − 4.051 × 10^−3^). The evolutionary rates of the 1st + 2nd codon positions (mean relative substitution rate 1.39, 95% HPD: 1.18–1.40) were significantly higher than that of the 3rd codon position (mean relative substitution rate 0.39, 95% HPD: 0.19–0.64).

### 3.3. Phylogenetic Analysis of the H7N9 PB1-F2 Gene

**Figure 2:**
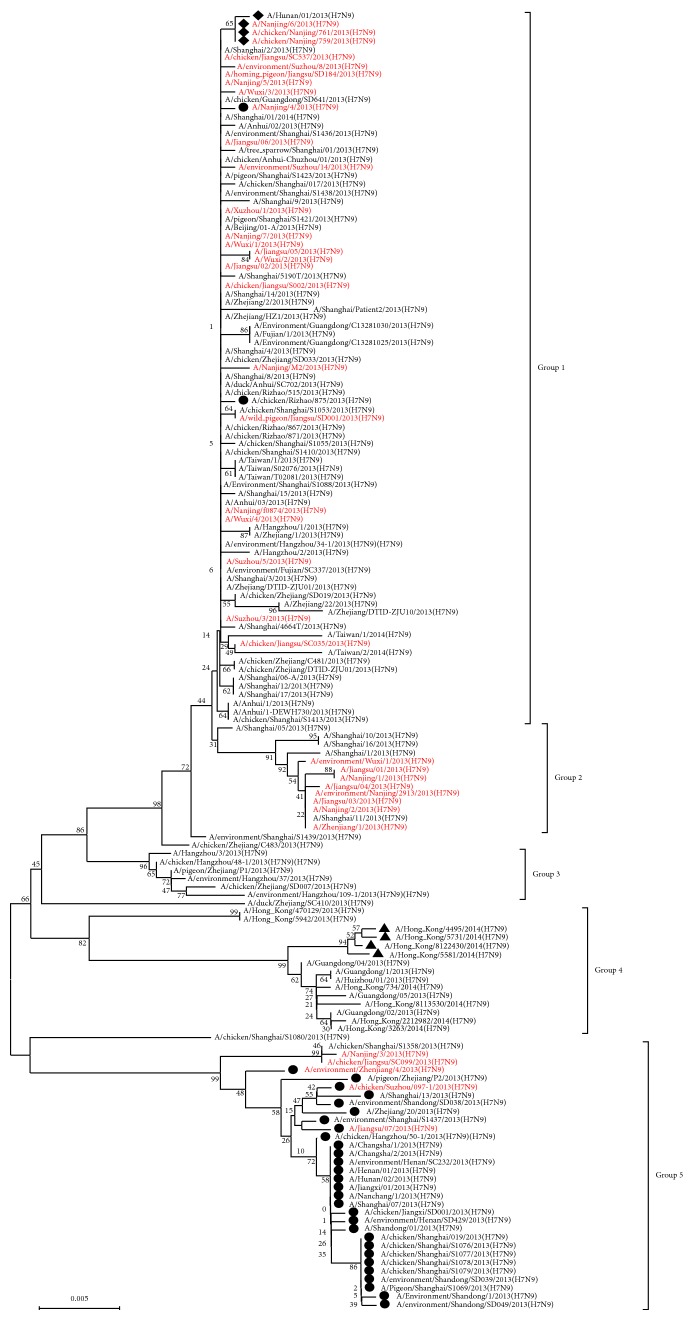
Phylogenetic tree of influenza A viruses for their PB1 gene nucleotide sequences. Sequence analysis was conducted using Molecular Evolutionary Genetics Analysis software by the neighbor-joining method. Note: ●: N-terminally truncated PB1-F2 (52aa); ◆: C-terminally truncated PB1-F2 (76aa); ▲: C-terminally truncated PB1-F2 (57aa).

38 Jiangsu H7N9 strains and 117 reference strains were included for the PB1 sequence analysis (Figure 2). The sequences of the H7N9 PB1-F2 were divided into five main lineages. The Jiangsu H7N9 strains were distributed in clusters 1, 2, and 5, were marked with red color, respectively. Most of the isolates contained an alternative start codon (AUG) at position 95 in the PB1 gene with a stop codon (UAG) at position 367, which encodes an intact PB1-F2 (90aa). 31 strains contained an alternative start codon (AUG) at position 209 in the PB1 gene, which translated into Met (M) and marked the beginning of the PB1-F2 ORF. Two strains were distributed in cluster 1 and 29 strains were distributed in cluster 5. These strains encountered a stop codon (UAG) at position 367 and encoded an N-terminal truncated ORF of 52 residues. A/Nanjing/6/2013, A/Hunan/01/2013, A/Chicken/Nanjing/759/2013, and A/Chicken/Nanjing/761/2013 of cluster 1 encountered a stop codon (UAG) at position 231, resulting in the production of a 76-residue peptide with only a C-terminally truncated PB1-F2. Four Hong Kong isolates (A/Hong_Kong/4495/2014, A/Hong_Kong/5581/2014, A/Hong_Kong/5731/2014, and A/Hong_Kong/8122430/2014) of cluster 4 encountered a stop codon (UAG) at position 174, resulting in the production of a 57-residue peptide with only a C-terminally truncated PB1-F2.

### 3.4. Amino Acid Analysis in H7N9 PB1-F2 Sequences

**Figure 3:**
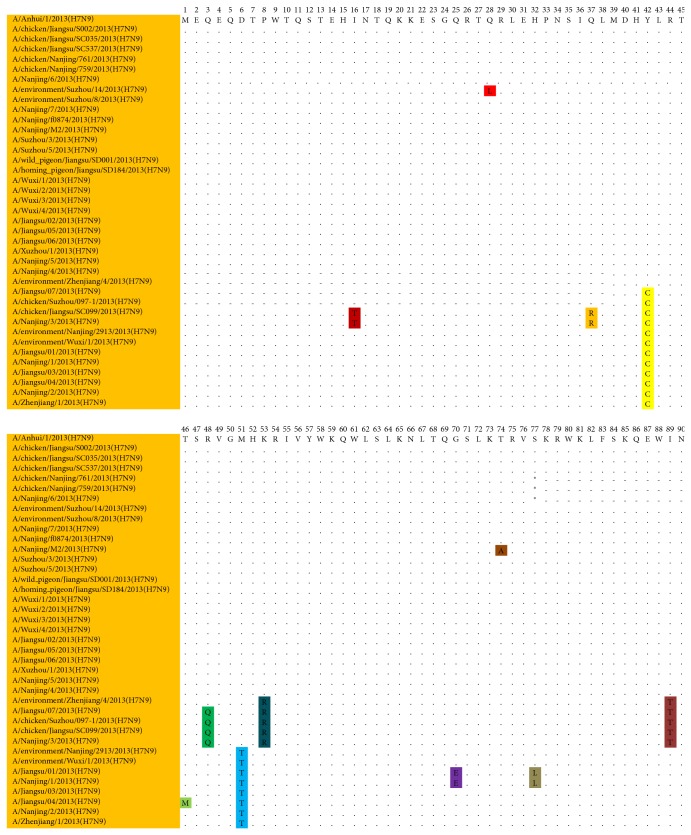
Alignment of putative PB1-F2 amino acid sequences of 38 Jiangsu H7N9 strains and A/Anhui/1/2013.

There are 12 positions differences between Jiangsu H7N9 isolates and A/Anhui/1/2013. Among these amino acid substitutions 6 positions located in the N-terminal and 6 positions located in the C-terminal, respectively. The Jiangsu H7N9 isolates of cluster 2 exhibited four amino acid substitutions, Y42C, R48Q, K53R, and I89T. There are two amino acid substitutions, Y42C and M51T, of all Jiangsu isolates of cluster 3, as shown in Figure 2. The pairwise identities between the Jiangsu H7N9 strains and A/Anhui/1/2013 were 96.5%–100% for nucleotides and 91.8%–100% for the translated amino acids. The pairwise identities among the Jiangsu H7N9 strains were 95.5%–100% for nucleotides and 88.9%–100% for the translated amino acids. The C-terminal portion of the protein, which includes the five amino acids 62–82 (L62, S66, R75, R79, and L82) using PB1-F2 (90aa) numbering, has been considered to be related to pathogenicity in the H3N2 and H5N1 backgrounds [21, 22]. The S66 polymorphism was not found in any of the 38 sequences. All of the sequences had four of the previous amino acids (Figure 3).

### 3.5. Selection Pressure

The selection pressures acting on the PB1-F2 gene of H7N9 and H9N2 viruses were estimated on the basis of the dN/dS ratio to have a mean value of 2.11. The site-by-site analysis of selection pressure analysis of PB1-F2 protein of 99 H7N9 and H9N2 virus strains revealed 12 positively selected sites and 3 negatively selected sites (16, 24, and 65) (Table 1). Among 12 positively selected sites identified in the PB1-F2 protein, sites 21, 30, 37, 38 43, 46, and 48 are located in the N-terminal; sites 54, 64, 72, and 82 are located in the C-terminal.

## 4. Discussion

According to the report by Fang et al., most human cases were concentrated at the Yangtze River Delta on China's eastern seaboard [23], and as we all know this region has large populations of humans, poultry, and pigs, with close contact between them. This may provide good opportunities for the H7N9 virus to adapt itself to mammals and reassort with other endemic human-adapted influenza viruses or pig-adapted influenza viruses. After closure of live poultry markets (LPM) human cases greatly decreased or disappeared, which suggests that LPM exposure plays a major role in human risk of avian influenza A H7N9 virus infection in urban areas [20]. But only 77% have a history of avian contact [1], and there is no evidence to support that human-to-human transmission has occurred, so the question is as follows: what contributes to the infection in cases with no history of avian or patient contact? Maybe a hidden epidemic in animals has well been underway. It is therefore critical to continue to conduct and strengthen both epidemiological and laboratory-based surveillance in both humans and animals of the H7N9 virus. The genetic mutation of the H7N9 virus should be followed closely and studied further, in order find out the epidemiological and molecular characteristics of H7N9 PB1-F2 in Jiangsu province.

The H7N9 virus was a new subtype with the 6 internal genes coming from the avian influenza A (H9N2) virus. In this study, we compared the H7N9 PB1-F2 with H9N2 PB1-F2 at the amino acid level. The evolutionary rates for PB1-F2 of H7N9 and H9N2 are identified as 3.053 × 10^−3^ substitutions per site per year, higher than the result of previous study of H3N8 [22], indicating that PB1-F2 of H7N9 might be in higher evolution pressure. In addition, it is interesting that usually we would expect the 3rd codon position to evolve faster; however, in our study the evolutionary rates of the 1st + 2nd codon positions were significantly higher than that of the 3rd codon position. Meanwhile, 12 positively selected sites and 3 negatively selected sites were confirmed by the estimate of site-by-site selection pressure analysis and this could explain why the nonsynonymous (1st + 2nd codon position) rates were higher than the synonymous one (3rd codon position). The present results reported are not consistent with earlier reports [24]. Usually in the early stages of viral pandemic outbreaks, the virus adapting into a new host will be faced with higher selection pressures; particularly, codons/amino acid sites of the virus under positive selection pressures are likely to be more [25]. The result of selection pressure analysis suggests positively selected sites may be useful for identifying the pathogenicity of the virus. We also compared the PB1-F2 amino acid sequences of 38 Jiangsu H7N9 strains and A/Anhui/1/2013. There are five major amino acid substitutions, Y42C, R48Q, M51T, K53R, and I89T. Amino acid substitutions suggest that the novel A/H7N9 virus with genomic segments from different subtypes/lineages has undergone dramatic changes in order to adapt to humans.

PB1-F2 protein is expressed by almost all of the avian influenza A strains; and the majority of the strains isolates express a full-length PB1-F2 protein. However, some strains from human and swine hosts have forms with either the C- or N-terminal truncations [26]. Since 1949, most H1N1 virus strains have had an incomplete PB1-F2 protein with truncation, most human H1N1 isolates have had a C-terminal truncated PB1-F2 (57aa), and the recent 2009 pandemic H1N1 virus encoded 11 amino acids with C-terminally truncated protein, which is thought to be lost functional. Perhaps this is the reason why infections over the last three decades with H1N1 strains have caused less morbidity and mortality than H3N2 strains [27–29]. Interestingly, recent H7N9 isolates contain an N-terminally truncated PB1-F2 (52aa) and a C-terminally truncated PB1-F2 (57aa and 76aa), which possibly plays a role in their decreased virulence. And we found that 25.2% (39/155) of the H7N9 isolates have truncated PB1-F2, which is higher than that of the H5N1 (2.7%) isolates according to the study of Smith and McCullers [21]. Košík and colleagues used plasmids expressing the N-terminal (1–50aa) or C-terminal (51–87aa) regions of the PB1-F2 molecule for transfection of MDCK cells coinfected with influenza A/Puerto Rico/8/34 (H1N1) virus deficient in PB1-F2 protein expression. They found that the N-terminal region of the PB1-F2 protein was responsible for increased PB1 protein expression [11].

The known functions of PB1-F2 are most associated with the C-terminal end of the protein. Yamada et al. concluded that the PB1-F2 residues in amino acids 46–75 were necessary and sufficient to form a mitochondrial targeting sequence (MTS) [30], and Gibbs et al. defined the minimal and the optimal MTS which ranges from amino acid 65 to amino acid 82 and 65 to 87 [31]. The MTS could affect PB1-F2 interactions with ANT3 and VDAC1 [6], potentially increasing the induction of apoptosis by PB1-F2. The amino acid residues L62, R75, R79, and L82 in the PB1-F2 proteins from epidemiologically important strains of 1918 (H1N1), 1957 (H2N2), 1968 (H3N2), and many highly pathogenic avian influenza viruses (H5N1) are contributors to cytokine release and inflammatory responses, with resulting pathological damage, weight loss, and death. However, this ability was lost in seasonal strains with either truncation (in the H1N1 lineage) or mutation [12, 22]. In our study, only 2 sequences were found that did not have R75 and 1 did not have R79, and the majority of the viruses had the previous four amino acids. The S66 was described as a factor affecting virulence and pathogenicity of IAV. And this position in 1918 IAV and HPAI HK97 was found to increase pathogenicity in mouse model. Similar immune suppression is seen when the mutation N66S is introduced to PB1-F2 in PR8 and H5N1 [32–34]. In our study the S66 amino acid was not found to be present in any of the 64 PB1-F2 sequences from H7N9 strains and this is consistent with the study of PB1-F2 in H5N1, where the S66 was uncommon [21]. Specific experiments probing the contribution of these amino acids in the H7N9 background are needed and require close surveillance.

## 5. Conclusions

The current research of influenza A (H7N9) virus was analyzed by using Bayesian Markov chain Monte Carlo evolutionary analyses, to investigate the epidemiological and molecular characteristics of the PB1-F2 proteins of novel influenza A (H7N9) virus in Jiangsu province, from March 1, 2013, to May 31, 2014. During February and March 2013, a novel influenza A (H7N9) virus occurred in Shanghai, China. And we found A (H7N9) virus has gained public health attention nowadays. Results have shown that 38 sequences of PB1 in H7N9 of Jiangsu were then divided into three lineages. Of these sequences, 4 sequences and 3 sequences encode an N-terminally truncated PB1-F2 (52aa) polypeptide and C-terminally truncated PB1-F2 (76aa) polypeptide, respectively. Whether the strains with N-terminally or C-terminally truncated PB1-F2 of H7N9 decrease the PB1 protein expression and virulence, and whether these strains would like H1N1 with truncated PB1-F2 will become the major epidemic strains, remains a question for further epidemiological surveillance. In addition we find a mean evolutionary rate of 3.053 × 10^−3^ subs/site/year. The site-by-site analysis of selection pressure analysis of PB1-F2 protein of 161 H7N9 and H9N2 virus strains revealed 12 positively selected sites and 3 negatively selected sites, indicating that PB1-F2 of H7N9 might be in higher evolution pressure. Meanwhile, the result of selection pressure analysis suggests positively selected sites, which may be useful for identifying the pathogenicity of the virus in the future.

## Supplementary Material

Accession numbers of Influenza A virus strain obtained form Global Initiative on Sharing Avian Influenza Data (GISAID) database, sources: , from January 1, 2013, and accessed on May 31, 2014.

## Figures and Tables

**Table 1:** Selection pressure analysis of PB1-F2 protein of H7N9 and H9N2 virus using SLAC, FEL, and REL methods.

Methods	Positive selection site data				Negative selection site data			
	Position	Normalised (*E*) dN/dS	*P* value	Bayes factor	Position	Normalised (*E*) dN/dS	*P* value	Bayes factor
SLAC	48	11.39	0.026	/	24	−7.98	0.039	/
					65	−9.36	0.032	/

FEL	21	53.25	0.028	/	24	−46.26	0.007	/
	37	25.16	0.097	/	65	−57.34	0.052	/
	46	27.01	0.091	/				
	48	77.94	0.003	/				
	54	33.57	0.042	/				
	82	30.98	0.039	/				

REL	30	2.43	/	83.30	16	−9.51	/	131.91
	38	2.44	/	113.39	24	−14.28	/	10558.2
	43	3.65	/	118.39	65	−12.57	/	11504.5
	48	18.14	/	357.09				
	64	2.38	/	200.36				
	72	3.71	/	198.05				
	82	4.75	/	187.46				

SLAC: single likelihood ancestor counting.

FEL: fixed effects likelihood.

REL: random effects likelihood.
