# Supplementary material for: Epidemiological and Molecular Characteristics of the PB1-F2 Proteins in H7N9 Influenza Viruses, Jiangsu
Source: Biomed Res Int. 2015 Jan 20;2015:804731. doi: 10.1155/2015/804731 (PMC4310227; doi:10.1155/2015/804731)
Supplement: Supplementary file 1 — Accession numbers of Influenza A virus strain obtained form Global Initiative on Sharing Avian Influenza Data (GISAID) database, sources: , from January 1, 2013, and accessed on May 31, 2014. [file 804731.f1.pdf]

| Accession | Virus name |
| --- | --- |
| CY187624 | Influenza A virus (A/Anhui/1-DEWH730/2013(H7N9)) |
| KF420308 | Influenza A virus (A/Changsha/1/2013(H7N9)) |
| KF420309 | Influenza A virus (A/Changsha/2/2013(H7N9)) |
| KF061024 | Influenza A virus (A/Fujian/1/2013(H7N9)) |
| KF944959 | Influenza A virus (A/Guangdong/02/2013(H7N9)) |
| KJ023072 | Influenza A virus (A/Guangdong/04/2013(H7N9)) |
| KJ023080 | Influenza A virus (A/Guangdong/05/2013(H7N9)) |
| KF662945 | Influenza A virus (A/Guangdong/1/2013(H7N9)) |
| KF001508 | Influenza A virus (A/Hangzhou/1/2013(H7N9)) |
| KF952512 | Influenza A virus (A/Hong Kong/470129/2013(H7N9)) |
| KF667737 | Influenza A virus (A/Huizhou/01/2013(H7N9)) |
| KF469232 | Influenza A virus (A/Nanchang/1/2013(H7N9)) |
| KC896772 | Influenza A virus (A/Nanjing/1/2013(H7N9)) |
| KF007042 | Influenza A virus (A/Nanjing/2/2013(H7N9)) |
| KF007050 | Influenza A virus (A/Nanjing/4/2013(H7N9)) |
| KF007114 | Influenza A virus (A/Nanjing/6/2013(H7N9)) |
| KF007146 | Influenza A virus (A/Nanjing/7/2013(H7N9)) |
| KJ411981 | Influenza A virus (A/Shanghai/01/2014(H7N9)) |
| KC853230 | Influenza A virus (A/Shanghai/4664T/2013(H7N9)) |
| KF997837 | Influenza A virus (A/Shanghai/5190T/2013(H7N9)) |
| KF007106 | Influenza A virus (A/Suzhou/3/2013(H7N9)) |
| KF007138 | Influenza A virus (A/Suzhou/5/2013(H7N9)) |
| KF018038 | Influenza A virus (A/Taiwan/1/2013(H7N9)) |
| KF018043 | Influenza A virus (A/Taiwan/S02076/2013(H7N9)) |
| KF018051 | Influenza A virus (A/Taiwan/T02081/2013(H7N9)) |
| KF034909 | Influenza A virus (A/Wuxi/1/2013(H7N9)) |
| KF034917 | Influenza A virus (A/Wuxi/2/2013(H7N9)) |
| KF007066 | Influenza A virus (A/Wuxi/3/2013(H7N9)) |
| KF007074 | Influenza A virus (A/Wuxi/4/2013(H7N9)) |
| KF150614 | Influenza A virus (A/Xuzhou/1/2013(H7N9)) |
| KJ633806 | Influenza A virus (A/Zhejiang/DTID-ZJU01/2013(H7N9)) |
| KC885961 | Influenza A virus (A/Zhejiang/DTID-ZJU01/2013(H7N9)) |
| KF732002 | Influenza A virus (A/Zhejiang/DTID-ZJU10/2013(H7N9)) |
| KF055468 | Influenza A virus (A/Zhejiang/HZ1/2013(H7N9)) |
| KF007058 | Influenza A virus (A/Zhenjiang/1/2013(H7N9)) |
| CY146906 | Influenza A virus (A/chicken/Guangdong/SD641/2013(H7N9)) |

| | |
|---|---|
| CY146914 | Influenza A virus (A/chicken/Jiangsu/S002/2013(H7N9)) |
| CY146922 | Influenza A virus (A/chicken/Jiangsu/SC035/2013(H7N9)) |
| CY146930 | Influenza A virus (A/chicken/Jiangsu/SC099/2013(H7N9)) |
| CY146938 | Influenza A virus (A/chicken/Jiangsu/SC537/2013(H7N9)) |
| CY146946 | Influenza A virus (A/chicken/Jiangxi/SD001/2013(H7N9)) |
| KF260709 | Influenza A virus (A/chicken/Rizhao/515/2013(H7N9)) |
| KF260710 | Influenza A virus (A/chicken/Rizhao/867/2013(H7N9)) |
| KF260711 | Influenza A virus (A/chicken/Rizhao/871/2013(H7N9)) |
| KF260712 | Influenza A virus (A/chicken/Rizhao/875/2013(H7N9)) |
| KF542888 | Influenza A virus (A/chicken/Shanghai/017/2013(H7N9)) |
| KF542889 | Influenza A virus (A/chicken/Shanghai/019/2013(H7N9)) |
| CY146954 | Influenza A virus (A/chicken/Shanghai/S1053/2013(H7N9)) |
| CY146962 | Influenza A virus (A/chicken/Shanghai/S1055/2013(H7N9)) |
| CY146970 | Influenza A virus (A/chicken/Shanghai/S1076/2013(H7N9)) |
| CY146978 | Influenza A virus (A/chicken/Shanghai/S1077/2013(H7N9)) |
| CY146986 | Influenza A virus (A/chicken/Shanghai/S1078/2013(H7N9)) |
| CY146994 | Influenza A virus (A/chicken/Shanghai/S1079/2013(H7N9)) |
| CY147002 | Influenza A virus (A/chicken/Shanghai/S1080/2013(H7N9)) |
| CY147010 | Influenza A virus (A/chicken/Shanghai/S1358/2013(H7N9)) |
| CY147018 | Influenza A virus (A/chicken/Shanghai/S1410/2013(H7N9)) |
| CY147026 | Influenza A virus (A/chicken/Shanghai/S1413/2013(H7N9)) |
| KF042095 | Influenza A virus (A/chicken/Zhejiang/C481/2013(H7N9)) |
| KF042096 | Influenza A virus (A/chicken/Zhejiang/C483/2013(H7N9)) |
| KC899667 | Influenza A virus (A/chicken/Zhejiang/DTID-ZJU01/2013(H7N9)) |
| CY147034 | Influenza A virus (A/chicken/Zhejiang/SD007/2013(H7N9)) |
| CY147042 | Influenza A virus (A/chicken/Zhejiang/SD019/2013(H7N9)) |
| CY147050 | Influenza A virus (A/chicken/Zhejiang/SD033/2013(H7N9)) |
| CY147058 | Influenza A virus (A/duck/Anhui/SC702/2013(H7N9)) |
| CY147066 | Influenza A virus (A/duck/Zhejiang/SC410/2013(H7N9)) |
| CY147074 | Influenza A virus (A/environment/Fujian/SC337/2013(H7N9)) |
| CY147082 | Influenza A virus (A/environment/Henan/SC232/2013(H7N9)) |
| CY147090 | Influenza A virus (A/environment/Henan/SD429/2013(H7N9)) |
| KC896769 | Influenza A virus (A/environment/Nanjing/2913/2013(H7N9)) |
| CY147098 | Influenza A virus (A/environment/Shandong/SD038/2013(H7N9)) |
| CY147106 | Influenza A virus (A/environment/Shandong/SD039/2013(H7N9)) |
| CY147114 | Influenza A virus (A/environment/Shandong/SD049/2013(H7N9)) |

| | |
|---|---|
| CY147122 | Influenza A virus (A/environment/Shanghai/S1088/2013(H7N9)) |
| CY147130 | Influenza A virus (A/environment/Shanghai/S1436/2013(H7N9)) |
| CY147138 | Influenza A virus (A/environment/Shanghai/S1437/2013(H7N9)) |
| CY147146 | Influenza A virus (A/environment/Shanghai/S1438/2013(H7N9)) |
| CY147154 | Influenza A virus (A/environment/Shanghai/S1439/2013(H7N9)) |
| KF034885 | Influenza A virus (A/environment/Suzhou/14/2013(H7N9)) |
| KF007002 | Influenza A virus (A/environment/Suzhou/8/2013(H7N9)) |
| KF150606 | Influenza A virus (A/environment/Wuxi/1/2013(H7N9)) |
| KF007010 | Influenza A virus (A/environment/Zhenjiang/4/2013(H7N9)) |
| CY147162 | Influenza A virus (A/homing pigeon/Jiangsu/SD184/2013(H7N9)) |
| CY147170 | Influenza A virus (A/pigeon/Shanghai/S1069/2013(H7N9)) |
| CY147178 | Influenza A virus (A/pigeon/Shanghai/S1421/2013(H7N9)) |
| CY147186 | Influenza A virus (A/pigeon/Shanghai/S1423/2013(H7N9)) |
| KF042093 | Influenza A virus (A/pigeon/Zhejiang/P1/2013(H7N9)) |
| KF042094 | Influenza A virus (A/pigeon/Zhejiang/P2/2013(H7N9)) |
| KJ508888 | Influenza A virus (A/tree sparrow/Shanghai/01/2013(H7N9)) |
| CY147194 | Influenza A virus (A/wild pigeon/Jiangsu/SD001/2013(H7N9)) |
| EPI447762 | Influenza A virus (A/Shanghai/8/2013(H7N9)) |
| EPI531801 | Influenza A virus (A/Taiwan/2/2014(H7N9)) |
| EPI447822 | Influenza A virus (A/Shanghai/06-A/2013(H7N9)) |
| EPI453648 | Influenza A virus (A/Nanjing/M2/2013(H7N9)) |
| EPI477408 | Influenza A virus (A/Zhejiang/22/2013(H7N9)) |
| EPI460749 | Influenza A virus (A/Nanjing/f0874/2013(H7N9)) |
| EPI443032 | Influenza A virus (A/Zhejiang/1/2013(H7N9)) |
| EPI447777 | Influenza A virus (A/Shanghai/14/2013(H7N9)) |
| EPI443040 | Influenza A virus (A/Zhejiang/2/2013 (H7N9)) |
| EPI447685 | Influenza A virus (A/Jiangsu/05/2013(H7N9)) |
| EPI447850 | Influenza A virus (A/Jiangsu/04/2013(H7N9)) |
| EPI447876 | Influenza A virus (A/Jiangsu/02/2013(H7N9)) |
| EPI447769 | Influenza A virus (A/Shanghai/17/2013(H7N9)) |
| EPI447678 | Influenza A virus (A/Jiangsu/06/2013(H7N9)) |
| EPI447699 | Influenza A virus (A/Hunan/01/2013(H7N9)) |
| EPI447960 | Influenza A virus (A/Shanghai/3/2013 (H7N9)) |
| EPI443570 | Influenza A virus (A/environment/Hangzhou/34-1/2013(H7N9) ) |
| EPI490969 | Influenza A virus (A/Environment/Guangdong/C13281025/2013(H7N9)) |

| | |
|---|---|
| EPI446450 | Influenza A virus (A/Hangzhou/2/2013(H7N9)) |
| EPI447741 | Influenza A virus (A/Shanghai/15/2013(H7N9)) |
| CY147162 | Influenza A virus (A/homing pigeon/Jiangsu/SD184/2013(H7N9)) |
| EPI439501 | Influenza A virus (A/Shanghai/2/2013(H7N9)) |
| EPI531795 | Influenza A virus (A/Taiwan/1/2014(H7N9)) |
| EPI447643 | Influenza A virus (A/Anhui/02/2013(H7N9)) |
| EPI447636 | Influenza A virus (A/Anhui/03/2013(H7N9)) |
| EPI439508 | Influenza A virus (A/Anhui/1/2013(H7N9)) |
| EPI447836 | Influenza A virus (A/Beijing/01-A/2013(H7N9)) |
| EPI447889 | Influenza A virus (A/chicken/Anhui-Chuzhou/01/2013(H7N9)) |
| EPI443657 | Influenza A virus (A/chicken/Hangzhou/48-1/2013(H7N9)) |
| EPI443665 | Influenza A virus (A/chicken/Hangzhou/50-1/2013(H7N9)) |
| EPI515776 | Influenza A virus (A/Chicken/Nanjing/759/2013(H7N9)) |
| EPI515786 | Influenza A virus (A/Chicken/Nanjing/761/2013(H7N9)) |
| EPI515794 | Influenza A virus (A/Chicken/Suzhou/097-1/2013(H7N9)) |
| EPI490977 | Influenza A virus (A/Environment/Guangdong/C13281030/2013(H7N9)) |
| EPI443673 | Influenza A virus (A/environment/Hangzhou/109-1/2013(H7N9)) |
| EPI443649 | Influenza A virus (A/environment/Hangzhou/37/2013(H7N9)) |
| EPI447650 | Influenza A virus (A/Environment/Shandong/1/2013(H7N9)) |
| EPI446456 | Influenza A virus (A/Hangzhou/3/2013(H7N9)) |
| EPI447727 | Influenza A virus (A/Henan/01/2013(H7N9)) |
| EPI457643 | Influenza A virus (A/homing_pigeon/Jiangsu/SD184/2013(H7N9)) |
| EPI507085 | Influenza A virus (A/Hong Kong/3263/2014 (H7N9)) |
| EPI502371 | Influenza A virus (A/Hong Kong/2212982/2014 (H7N9)) |
| EPI490880 | Influenza A virus (A/Hong Kong/5942/2013 (H7N9)) |
| EPI498798 | Influenza A virus (A/Hong Kong/734/2014(H7N9)) |
| EPI516604 | Influenza A virus (A/Hong Kong/8122430/2014 (H7N9)) |
| EPI520862 | Influenza A virus (A/Hong Kong/5731/2014 (H7N9)) |
| EPI516596 | Influenza A virus (A/Hong Kong/5581/2014 (H7N9)) |
| EPI509878 | Influenza A virus (A/Hong_Kong/4495/2014(H7N9)) |
| EPI509886 | Influenza A virus (A/Hong Kong/8113530/2014 (H7N9)) |
| EPI447692 | Influenza A virus (A/Hunan/02/2013(H7N9)) |
| EPI460765 | Influenza A virus (A/Nanjing/3/2013(H7N9)) |
| EPI460773 | Influenza A virus (A/Nanjing/5/2013(H7N9)) |
| EPI447920 | Influenza A virus (A/Jiangsu/01/2013(H7N9)) |
| EPI447876 | Influenza A virus (A/Jiangsu/02/2013(H7N9)) |

| | |
|---|---|
| EPI447863 | Influenza A virus (A/Jiangsu/03/2013(H7N9)) |
| EPI447850 | Influenza A virus (A/Jiangsu/04/2013(H7N9)) |
| EPI447685 | Influenza A virus (A/Jiangsu/05/2013(H7N9)) |
| EPI447678 | Influenza A virus (A/Jiangsu/06/2013(H7N9)) |
| EPI447671 | Influenza A virus (A/Jiangsu/07/2013(H7N9)) |
| EPI447706 | Influenza A virus (A/Jiangxi/01/2013(H7N9)) |
| EPI447720 | Influenza A virus (A/Shandong/01/2013(H7N9)) |
| EPI447910 | Influenza A virus (A/shanghai/05/2013(H7N9)) |
| EPI447897 | Influenza A virus (A/Shanghai/07/2013(H7N9)) |
| EPI439489 | Influenza A virus (A/Shanghai/1/2013(H7N9)) |
| EPI447809 | Influenza A virus (A/Shanghai/10/2013(H7N9)) |
| EPI447801 | Influenza A virus (A/Shanghai/11/2013(H7N9)) |
| EPI447793 | Influenza A virus (A/Shanghai/12/2013(H7N9)) |
| EPI447784 | Influenza A virus (A/Shanghai/13/2013(H7N9)) |
| EPI447950 | Influenza A virus (A/Shanghai/4/2013(H7N9)) |
| EPI447950 | Influenza A virus (A/Shanghai/9/2013(H7N9)) |
| EPI451251 | Influenza A virus (A/Shanghai/Patient2/2013(H7N9)) |
| EPI477400 | Influenza A virus (A/Zhejiang/20/2013(H7N9)) |
| EPI477408 | Influenza A virus (A/Zhejiang/22/2013(H7N9)) |
